# Heat shock protein and heat shock factor 1 expression and localization in vaccinia virus infected human monocyte derived macrophages

**DOI:** 10.1186/1476-9255-2-12

**Published:** 2005-10-24

**Authors:** Aleksandra Kowalczyk, Krzysztof Guzik, Kinga Slezak, Jakub Dziedzic, Hanna Rokita

**Affiliations:** 1 Jagiellonian University, Faculty of Biotechnology; 7, Gronostajowa St., 30-387 Krakow, Poland

## Abstract

**Background:**

Viruses remain one of the inducers of the stress response in the infected cells. Heat shock response induced by vaccinia virus (VV) infection was studied in vitro in human blood monocyte derived macrophages (MDMs) as blood cells usually constitute the primary site of the infection.

**Methods:**

Human blood monocytes were cultured for 12 – 14 days. The transcripts of heat shock factor 1 (HSF1), heat shock protein 70 (HSP70), heat shock protein 90 (HSP90) and two viral genes (E3L and F17R) were assayed by reverse transcriptase-polymerase chain reaction (RT-PCR), and the corresponding proteins measured by Western blot. Heat shock factor 1 DNA binding activities were estimated by electrophoretic mobility shift assay (EMSA) and its subcellular localization analyzed by immunocytofluorescence.

**Results:**

It appeared that infection with vaccinia virus leads to activation of the heat shock factor 1. Activation of HSF1 causes increased synthesis of an inducible form of the HSP70 both at the mRNA and the protein level. Although HSP90 mRNA was enhanced in vaccinia virus infected cells, the HSP90 protein content remained unchanged. At the time of maximum vaccinia virus gene expression, an inhibitory effect of the infection on the heat shock protein and the heat shock factor 1 was most pronounced. Moreover, at the early phase of the infection translocation of HSP70 and HSP90 from the cytoplasm to the nucleus of the infected cells was observed.

**Conclusion:**

Preferential nuclear accumulation of HSP70, the major stress-inducible chaperone protein, suggests that VV employs this particular mechanism of cytoprotection to protect the infected cell rather than to help viral replication. The results taken together with our previuos data on monocytes or MDMs infected with VV or *S. aureus *strongly argue that VV employs multiple cellular antiapoptotic/cytoprotective mechanisms to prolong viability and proinflammatory activity of the cells of monocytic-macrophage lineage.

## Background

Manipulation of the immune system, especially interference with specific components of the apoptotic response of the infected cells is essential for a virus to replicate and to disseminate in a host.

Vaccinia virus belongs to the poxviruses super-family, a group of large DNA viruses known from their exclusive propagation outside the nucleus in the cytoplasm of the infected cell [1]. Vaccinia virus infections are commonly associated with a generalized host cell protein and nucleic acids synthesis inhibition, depending on time and an infectious dose. Despite the observed shutdown of host transcriptional and translational mechanisms and selective expression of many viral genes, several eukaryotic proteins are transiently induced or activated by poxviruses, e.g. transcription factors [2], cytokines [3,4], heat shock proteins [5] and antioxidant enzymes [6]. Moreover, although mainly necrotic, vaccinia virus is opposing the apoptosis due to several anti-apototic genes present and expressed from its genome [7,8].

Stress conditions like heat shock, infections, radiation, and exposure to chemicals induce increased levels of heat shock proteins in many cell lines [9]. The heat shock proteins can be induced in vitro following infection by a variety of viruses [10] such as Ad5 and HSV-1 which have been shown to induce synthesis of one of the main heat shock proteins, HSP70. Vaccinia virus was already found to be a potent inducer of HSP70 in mice [11,12]. The role of HSP70 in vaccinia virus infection has not been elucidated so far, however the results of the earlier studies in vaccinia virus infected U937 cells and primary macrophages suggest its role in viral protein folding and virus assembly [5]. Moreover, in vivo studies in mice reveal lack of the influence of infection on viral life cycle [12]. Obviously, HSPs constitute specific chaperons for the viral proteins necessary to secure proper folding, translocation and formation of multi-component complexes of the viral proteins. Recent investigations indicate that the heat shock proteins exert suppression of the apoptosis [13-15] and therefore might support vaccinia virus infection.

Induction of the heat shock protein synthesis requires earlier activation of heat shock factors. HSF1 is assumed to be the main mediator of the cellular stress response, which binds to the heat shock promoter element (HSE) [16,17]. It is believed that in normal conditions monomers of HSF1 exist inactive in the cytoplasm in large complexes with other heat shock proteins, e.g. HSP90, and HSP70. Upon stress, when the heat shock proteins are needed, HSF1 undergoes trimerization, subsequent translocation into the nucleus and binding to the heat shock elements within the regulatory sequences of the heat shock protein genes [15,18].

To understand further the role of vaccinia virus in the course of the infection, the heat shock response was studied in human blood monocyte derived macrophages infected with the vaccinia virus Western Reserve strain.

## Methods

### Cell culture

Peripheral blood leukocytes (PBL) were isolated by standard Ficoll-Paque (Pharmacia, Uppsala, Sweden) gradient centrifugation from the blood of healthy donors. The cells were cultured at the concentration of 2 × 10^7 ^of PBL cells per 5.5 cm dish for protein harvesting or at the concentration of 8 × 10^6 ^of PBL cells per 3.5 cm dish for immunocytochemical analyses. The cells were cultured for 10–14 days in RPMI medium (Gibco) with 10% human serum (AB serotype); medium was changed every 48 hours until the monocytes reached adherence. The adherent monocytes constitute 10 % of the total PBLs placed on the dish. Human hepatoma HepG2 cells were cultured in DMEM with 10% FCS in 60 mm-diameter culture dishes for 48 hours before infection or heat shock.

### Virus propagation

The vaccinia virus Western Reserve strain was propagated in VERO-B4 cells (DSMZ, Germany) infected at multiplicity of infection (MOI) of 1 (one plaque forming unit, pfu, per cell) maintained in MEM supplemented with 4% heat-inactivated FCS. Infected cells were harvested when the maximum cytopathic effect was observed and infectivity was estimated by a quantal infectivity assay on VERO-B4 cells [19] and a standard plaque assay. Human macrophages were infected with the virus at multiplicity of infection 1 or 5. Infected cells were washed after 1 h of virus adsorption and fresh medium added.

### Heat shock

The heat shock was performed in 42°C in the water bath for one hour, followed by 2–4 hours recovery at 37°C.

### Protein isolation

The cells were washed with 1 ml cold PBS and harvested to the Eppendorf 2 ml tubes in 1–2 ml of PBS. The harvested cells were centrifuged at 250 × g for 5 min. The cell pellet was suspended either in 400 μl of the resuspension buffer for isolation of the nuclear and cytoplasmic fractions of proteins according to the Suzuki method [20], or in 150 μl of the extraction buffer (50 mM Tris pH 8.0, 10 mM CHAPS, 2 mM EDTA, 1 mM Na_3_VO_4_, 5 mM DTT, 1 mM PMSF, 10% glycerol) for whole cell extracts [21]. According to the Suzuki method, the nuclear fraction contains all nuclei, and the remaining supernatant is termed "cytoplasmic fraction". Contamination of the nuclei with cytoplasm was excluded based on lactate dehydrogenase activity measurements using a LDH detection kit from Boehringer Mannheim.

Protein concentrations were measured using the BCA assay (Sigma) based on bicinchoninic acid [22]. The absorbance was measured at 562 nm in SpectraMax 250 microplate reader (Molecular Devices).

### Western blot

Equal amounts of protein extracts (10 μg/lane) were separated by SDS-PAGE according to the protocol described by Laemmli [23]. The protein transfer was performed in a semi-dry blotting system (Fastblot B31, Biometra) in the transfer buffer (25 mM Tris pH 8.3, 0.2 M glycine, 20% methanol, v/v) at 35 V for 30 min. Equal loading of samples, and even transfer, were confirmed by staining the membranes with Ponceau S. The membrane (Hybond, Amersham Pharmacia) was blocked with 5% powdered milk in TST buffer (10 mM Tris HCl pH 7.5, 0.9 % NaCl, 0.05 % Tween 20) for 1.5 h, followed by a 20-min wash in the TST buffer. The membrane was incubated either with the primary anti-HSF1 (H-311, sc-9144), or anti-HSP70 (K-20, sc-1060), or anti HSP90 antibodies (H-114, sc-7947) from Santa Cruz Biotechnology, in 1:1000 dilution in the TST buffer with 2 % BSA for 1 h. The membrane was then washed four times in TST buffer for 15 min. Secondary anti-rabbit IgG antibodies coupled to horseradish peroxidase (Amersham Pharmacia) were diluted 1:5000 in TST buffer with 2 % BSA. The membrane was incubated with the secondary antibodies for 1 hour, followed by four washes of 15 min. in TST buffer. The ECL-plus kit (Amersham Pharmacia) was used to visualize the protein. The membranes were exposed into X-ray films for 10 minutes to 1 hour, and the films were developed.

### Electrophoretic mobility shift assay

A DNA mobility shift assay was carried out as described by Duyao [24]. The double-stranded oligonucleotides containing the HSF binding site (5'-CTAGAAGCTTCTAGAAGCTTCTAGAA-3') were an "optimal" heat shock element (HSE) containing five perfect inverted nGAAn repeats from the human *hsp70 *[25]. DNA fragments were labeled using Klenow polymerase and [α-32P]dCTP by filling 5'-overhangs of four bases at both ends after annealing. Equal amounts of protein (5 μg) in 10% glycerol were incubated at room temperature for 30 min. with 0.5 ng of the labeled dsDNA oligonucleotide in the presence of 2 μg of poly(dI-dC) in 10 mM Tris pH 7.5, 50 mM NaCl, 1 mM EDTA and 0.1 mM DTT in a total volume of 20 μl. For supershift analysis, the rabbit polyclonal antibodies against human HSF1 (H-311, sc-9144X) from Santa Cruz Biotechnology were also preincubated with protein extracts in 1:20 dilutions. Incubation mixtures were electrophoresed on 4.5% nondenaturing polyacrylamide gel in 0.5 × TBE. The dried gels were analyzed by autoradiography.

### RNA extraction and RT-PCR

Total RNA was extracted from cultured cells using Trizol reagent (Gibco). RNA samples (2 μg) were used for cDNA synthesis reactions in a total volume of 20 μl containing 10 μl of each RNA sample, 0.5 μg oligo (dT)12–18 primer (Gibco) and 200 U of SuperScript II RNAse H-Reverse Transcriptase (Gibco) according to the protocol provided with the enzyme. Although some RNA samples were treated with RNase-free DNase to remove all genomic DNA prior to the RT reaction, similar results were received using DNase untreated RNA preparations. The PCR reactions were done using F105S Taq polymerase (Polygen) in the mixes containing: 5 μl 10 × PCR buffer, 1 μl 10 mM dNTPs, 2 μl of each primer, 50 mM KCl, 1.5 mM MgCl_2_, 2.5 U of (1 μl) Taq polymerase, 2 μl cDNA and 37 μl sterile water. Reactions were carried out at the following conditions: 94°C for 1 min, 60°C for 1 min, and 72°C for 1.5 min for 30 cycles (*hsp70 *and *hsf1*) or 95°C for 1 min, 50°C for 1 min, and 72°C for 1.5 min for 30 cycles (*hsp90*α) or 94°C for 1 min, 55°C for 1 min, and 72°C for 1.5 min for 30 cycles (*E3L*, *F17R*, and β-*actin*). Each thermal profile was ended with the final extension at 72°C for 15 min. The reaction products were then resolved on nondenaturing 2% agarose gel and visualized by staining with ethidium bromide. The primer sequences are listed in Table 1. The primers were designed to match sequences in separate exons (except for the *hsp70 *encoded by a single exon) to avoid the contribution of genome-templated product in the signal analysis.

**Table 1:** Sequences of the primers used in the RT-PCR reaction and the size of the amplified products of human and viral genes

**Gene of interest**	**Size of the product**	**Primer orientation**	**Primer sequence**
β-*actin*	307 bp	Forward Reverse	5' AGCGGGAAATCGTGCGTG 3' 5' GGGTACATGGTGGTGCCG 3'
*hsf1*	577 bp	Forward Reverse	5' ATGGCCAGCTTCGTGCG 3' 5' ACAGCATCAGGGGCGTA 3'
*hsp70*	590 bp	Forward Reverse	5' TTTGACAACAGGCTGGTGAACC 3' 5' GTGAAGGATCTGCGTCTGCTTGG 3'
*hsp90*	1498 bp	Forward Reverse	5' GCTGTGCCGTTGGTCCTGTGC 3' 5' GGTTCTCCTTCATTCTGGTGC 3'
*E3L*	360 bp	Forward Reverse	5' TATATTGACGAGAGTTCTGAC 3' 5' ACTCATTAATAATGGTGACAGG 3'
*F17R*	283 bp	Forward Reverse	5' ATTCTCATTTTGCATCTGCTC 3' 5' AGCTACATTATCGCGATTAGC 3'

### Immunofluorescence cell staining

The cells were cultured on sterile glass cover slips mounted in 3.5 cm culture dishes. Cells were fixed with 3% paraformaldehyde in PBS at 37°C for 15 min and permeabilized with 0.1% Triton-X-100 in PBS for 5 min at room temperature. Nonspecific binding sites were blocked with 3% bovine serum albumin solution in PBS and cells were stained with the anti-human HSF1 (H-311, sc-9144) rabbit polyclonal antibodies (Santa Cruz Biotechnology) in 1:200 dilution. The secondary sheep anti-rabbit Cy3 conjugated IgGs (Sigma, C2306) were used in 1:200 dilution. Nuclear DNA was additionally stained with Hoechst 33258 (Molecular Probes) at a concentration 0.5 μg/ml for 10 min at room temperature and then washed three times with PBS. Cover slips were mounted on microscopic glass slides using Vectashield (Vector Laboratories) to prevent fading of the fluorescent dye. Microphotographs were taken using a Leitz Orthoplan microscope with an epifluorescence and phase-contrast optics equipped with the Nikon FX-35DX camera on high sensitivity Kodak TMAX 3200 films. From one spot both, phase-contrast pictures as well as fluorescence pictures were taken.

## Results

### Changes in the heat shock factor 1 and the heat shock protein mRNAs content during vaccinia virus infection of human blood macrophages

**Figure 1:**
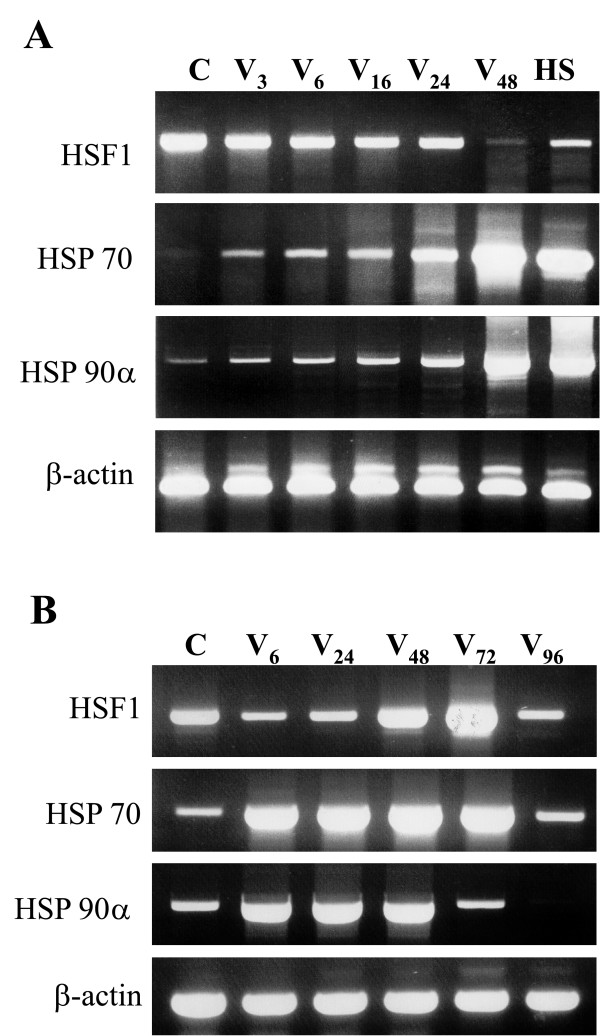
**RT-PCR analysis of the heat shock factor 1 and heat shock proteins in vaccinia virus infected human blood macrophages**. HSF1, HSP70, HSP90α and β-actin mRNAs were measured at 3, 6, 16, 24, 48, 72 and 96 hours p.i. PCR data come from a single representative experiment being one of three separate experiments using the cells from healthy donors. Vaccinia virus (V) infection was carried out at MOI 1 (A) and 5 (B), control (C). HS – heat shock at 42°C for 1 h plus recovery at 37°C for 2 h. β-actin gene product was used as a control.

The levels of HSF1, HSP70 and HSP90 mRNAs were determined by RT-PCR in control and virus-infected macrophages. In most unstressed cells, neither HSP70, nor HSP90 mRNA were detected (Fig. 1A), although in some cultures a basal level of both transcripts was visible (Fig. 1B). This heterogeneity was probably due to individual features of blood or serum donors. In contrary, HSF1 mRNA was constitutively accumulated in all examined cultures. The analysis showed no increase of the HSF1 transcripts up to 48 h p.i. (with a low infectious dose, 1 pfu/cell), similarly to the heat shock response when HSF1 mRNA is not induced (Fig. 1A). However, biphasic kinetics of HSF1 mRNA is observed after high dose infection (5 pfu/cell) with the minimum at 6 to 24 h p.i. corresponding to the maximal viral gene transcription (Fig. 1B) [5]. Subsequent decline in viral transcription was followed by increased HSF1 mRNA content.

HSP70 mRNA increased early upon infection with a high vaccinia virus dose, and clear decrease was found fairly late, at 96 hours p.i. The transcript increase after low infectious dose was slowly reaching the maximum at 48 h p.i. The kinetics of HSP90 mRNA increase upon high vaccinia virus dose was similar to that of HSP70 mRNA, however its level decreased earlier than the levels of HSP70 transcript, as this was observed already at 72 hours p.i. At MOI 1, HSP90 mRNA increase was slow, similar to the increase of HSP70 mRNA. HSP70, HSP90 and HSF1 transcripts estimated after the heat shock of the macrophages are also included for comparison and β-actin transcript is shown as a control.

### Viral gene expression in the macrophages

**Figure 2:**
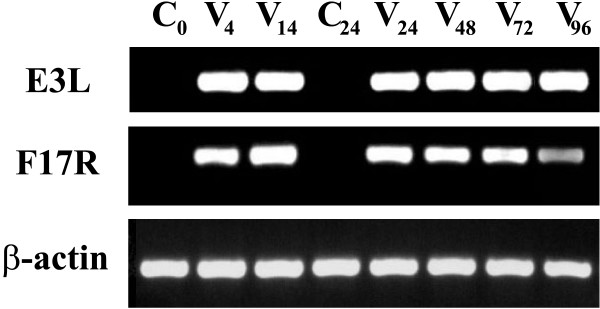
**Viral early (*E3L*) and late (*F17R*) genes expression in the infected human adherent monocytes**. Two vaccinia virus transcripts of *E3L *and *F17R *genes and of the cellular gene, β-actin, as a control, were estimated by RT-PCR. Representative results of three independent experiments are shown.

In order to check the viral infection itself, two viral genes were chosen: early gene *E3L*, responsible for the vaccinia virus antiapoptotic defence on the interferon pathway [26], and late viral gene, *F17R*, the product of which takes part in the mature virion assembly [27]. The RT-PCR of the viral genes showed an increased amount of E3L mRNA at 4 h p.i., which was maintained up to 96 h p.i. F17R mRNA was detected also at 4 h p.i. but increased at 14 h and maintained elevated up to 96 h p.i. (Fig. 2). The results evidenced, that late viral DNA replication had not been stopped in the macrophages. Moreover, increased and persistent levels of E3L mRNA support our conclusion on resistance to apoptosis elicited in the infected cells.

### HSF 1 protein activity and localization in the vaccinia virus infected macrophages

**Figure 3:**
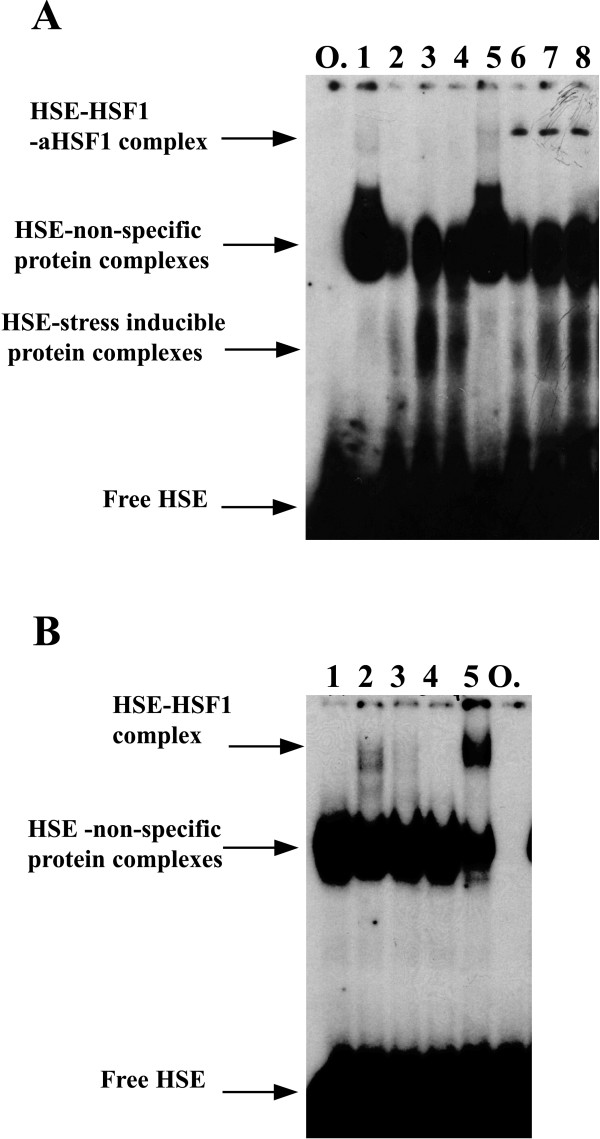
**Vaccinia virus-induced HSE binding activity in macrophages and a human hepatoma cell line**. (A) Macrophages were infected with vaccinia virus (V) with 5 pfu/cell and cultured for 16 or 24 hours (supershift assay). Lane 1 – NE from infected macrophages at 16 h p.i., lane 2 – WCE from control macrophages, lane 3 – WCE from infected macrophages at 16 h p.i., lane 4 – WCE from infected macrophages at 24 h p.i.; lanes 5–8 – as lanes 1–4 plus preincubation with 1:20 dilution of antibodies against HSF1 (aHSF1). (B) HepG2 cells (3 × 10^6^) were infected with vaccinia virus at MOI 1 for 24 h or heat shock treated (44°C, 20 min) or heat shock treated and recovered for 2 or 4 h at 37°C (shift assay). Lane 1 – control cells, lane 2 – heat shock treated, lane 3 – heat shock treated and recovered for 2 h, lane 4 – heat shock treated and recovered for 4 h, lane 5 – vaccinia virus infected for 24 h. O – ds oligoDNA (free HSE) incubated without proteins. Exposure time: 6 days (A) and 18 h (B). A single representative experiment being one of four separate experiments is shown.

Heat shock factor 1 DNA binding activity was analyzed in the nuclear and whole cell extracts of macrophages by an electrophoretic mobility shift assay. EMSA showed protein binding to the heat shock element in the control, at 16, and 24 hours of vaccinia virus infection (Fig. 3A). Supershift analysis of the whole cell extracts from vaccinia virus infected cells and the extracts from uninfected cells confirmed that heat shock factor 1 was present in equal quantities (Fig. 3A). However, nuclear proteins isolated at 16 h p.i. (Fig. 3A, lane 5) did not form the clear shifted antibody-HSE complex, suggesting that the epitopes recognized by the polyclonal antibodies are obscured by other proteins. Similar DNA-protein complex, without the antibodies against HSF1 added, was observed only in vaccinia virus-infected and heat shock treated human hepatoma HepG2 cell line (Fig. 3B).

**Figure 4:**
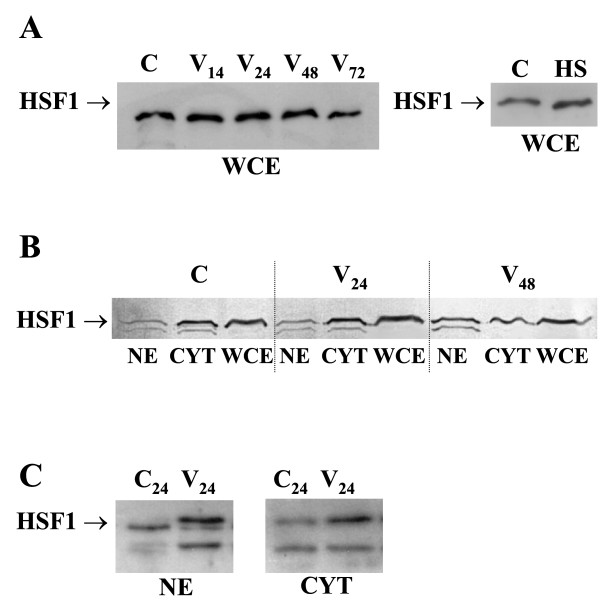
**Western blot analysis of subcellular localization of HSF1 in vaccinia virus infected macrophages**. Whole cell extracts (WCE) (A), nuclear extracts (NE) (B, C) and cytoplasmatic fraction(CYT)(B, C) of vaccinia virus infected macrophages were analysed by Western blot. C – control, HS – heat shock. Vaccinia virus (V) infection was carried out at MOI 5.

Although HSF1 protein content did not change in the whole cell extracts after vaccinia virus infection and during the heat shock response (Fig. 4A), more HSF1 accumulated in the nuclei of infected cells than in the nuclei of control cells, especially at 48 h p.i. (Fig. 4B and 4C) as Western blot analysis revealed. The analysis with a polyclonal anti-HSF1 serum shows two HSF1 bands with mobilities of approximately 70 and 80 kDa, which differ in phosphorylation state (Fig. 4C) [28]. The hyperphosphorylation of HSF1 and translocation of the factor into the nucleus of vaccinia virus-infected macrophages was clearly seen at 24 h p.i. (Fig. 4C).

**Figure 5:**
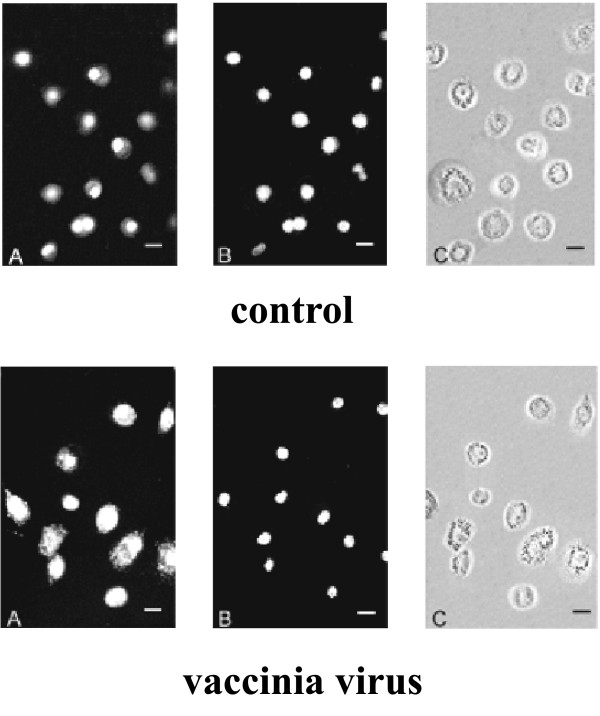
**Vaccinia virus-induced HSF1 redistribution**. Cells uninfected (control) or infected for 24 h (MOI 5) were fixed and allowed to react with anti-human HSF1 antibodies (A) or stained with Hoechst 33258 (B). Panels A, B – epifluorescence, C – phase-contrast picture of the same cells. Representative images of three independent experiments are shown. The inserted bar – 20 μm.

Indirect immunocytochemical staining of macrophages with anti-HSF1 antibodies (the secondary antibodies conjugated with Cy3) showed prevalent nuclear and weak cytoplasmic localization of the factor in the control cells (Fig. 5). Even more protein was observed in the nuclei and cytoplasm of vaccinia virus infected cells at 24 h p.i. The percentage of the cells containing HSF1 exclusively in their nuclei was calculated based on the immunocytochemical staining of the cells and mean values of at least 100 cells randomly selected on each sample were 24% and 46% for infected and control cells respectively.

### HSP70 protein increases during infection and transiently accumulates in the nucleus

**Figure 6:**
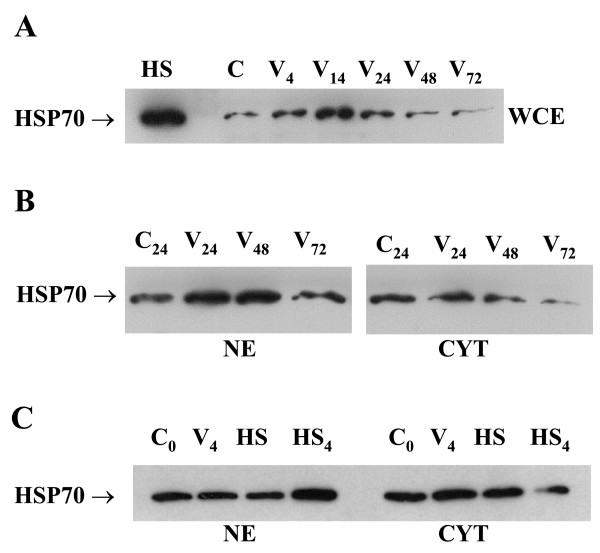
**Changes in HSP70 content in vaccinia virus infected macrophages**. Whole cell extracts (WCE) (A), nuclear extracts (NE) (B, C) and cytoplasmatic fraction (CYT) (B, C) of vaccinia virus infected macrophages were analysed by Western blot. C – control, HS – heat shock (proteins extracted after 4 hours recovery from heat shock). Vaccinia virus (V) infection was at MOI 5.

HSP70 protein content increased during the first 14 hours of infection (Fig. 6A) but no as much as it was shown for the heat shock treated macrophages. The increase reflected earlier changes in HSP70 mRNA content shown in Fig. 1. Prevalent nuclear accumulation of the protein was observed fairly late at 24 and 48 hours p.i. (Fig. 6B), while no change was found at 4 h p.i. (Fig. 6C). Data from the heat shock treated and the heat shock recovered cells are also included (Fig. 6C).

### HSP90 protein does not increase during infection but transiently locates in the nucleus

**Figure 7:**
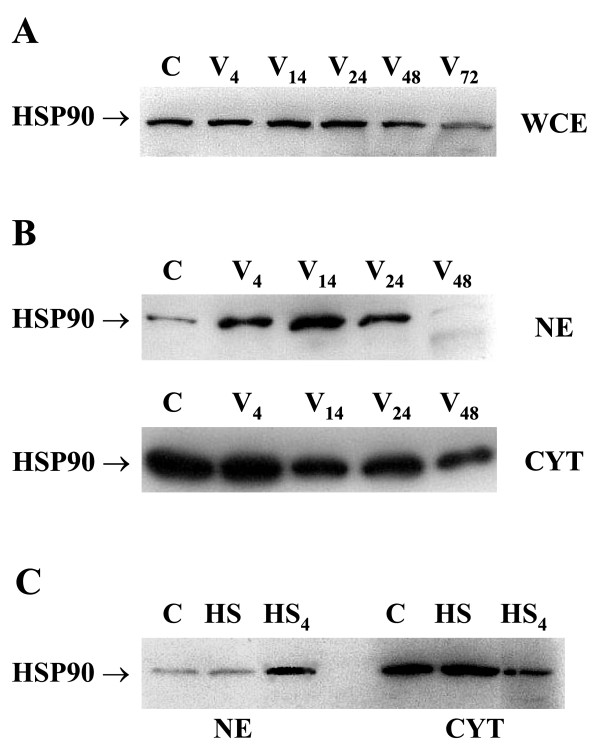
**HSP90 content in vaccinia virus infected macrophages**. Whole cell extracts (WCE) (A), nuclear extracts (NE) (B, C) and cytoplasmatic fraction (CYT) (B, C) of vaccinia virus infected macrophages were analysed by Western blot. C – control, HS – heat shock, HS_4 _– heat shock and 4 h recovery. Vaccinia virus (V) infection was at MOI 5.

HSP90 protein content did not change during in vitro infection as estimated by Western blot in the whole cell extracts (Fig. 7A). However, early (at 14 hours p.i.) increase in the nuclear content of the protein was found similarly to the results obtained for HSP70 protein (Fig. 7B). Additional analysis of HSP90 content in the heat shock treated and the heat shock recovered macrophages revealed that the heat shock similarly to the vaccinia virus infection, cause HSP90 protein translocation into the nucleus and the effect was clearly seen 4 hours after the heat shock (Fig. 7C).

## Discussion

Several cellular proteins are used by poxviruses and one of the examples is HSP70, which aggregates with viral proteins in the cytoplasm [5]. Although vaccinia virus life cycle does not appear to depend on HSP70 expression [12], the HSP70 transcripts as well as the protein increase significantly in human macrophages at 4 to 24 h p.i., as shown by us and others [5]. It has been well documented that protection against stress-induced apoptosis depends on the chaperone function of HSP70 [14]. Therefore, the results presented in this study indicate that HSP70 might be one of the factors responsible for the survival of VV-infected macrophages. In contrast to the results presented by others we also suggest a more important role of the nuclear pool of HSP70 [5]. Our data showing the predominant nuclear localization of HSP70 do not support the hypothesis on the possible role of HSP70 in folding of viral proteins [5], but speak rather in favor of its protective role in biogenesis of ribosomes within the nucleoli of the infected cells [29].

The role of HSP90 in viral infection, especially its nuclear accumulation (Fig. 7), remains unclear. Our earlier studies [4] have already revealed the stimulatory effect of the vaccinia virus infection on IL-10 gene expression in human blood elutriated monocytes. The finding stays in agreement with the data on the enhancement of the *hsp90 *gene expression by IL-10 in a human hepatoma HepG2 cell line and peripheral blood mononuclear cells [30]. The lack of HSP90 protein induction in the vaccinia virus infected cells was already found by others [10,31]. However, these authors [10] failed to detect an increased induction of HSP70, and this observation stays in contrast to our results (Fig. 6). Moreover, differential kinetics of HSP70 and HSP90 mRNA levels following exposure to a heat shock in human blood adherent monocytes was also found [32], therefore the heat shock response seems to be similar in this aspect to the vaccinia virus infection.

HSF1 is not a stress-inducible protein, neither is its expression level coupled to the rate of expression of the heat shock genes [33]. Although the vaccinia virus infection causes transient increase of HSF1 mRNA, no increase in HSF1 protein content is found, probably due to the instability of its mRNA. On the other hand, the decrease in HSF1 mRNA observed at the beginning of the infection does not severely affect the protein content because of a fairly long half life time of HSF1 protein [15]. The small decrease in HSF1 content found by us on the third day p.i. (Fig. 4A), might result from limited cellular protein synthesis observed during the prolonged viral infection.

In resting cells, HSF1 is predominantly found in a diffuse cytoplasmic and nuclear distribution, and after the heat shock it relocates rapidly to form large and irregularly shaped nuclear granules [34]. These nuclear structures, referred to as the HSF1 stress granules, can be induced by various stresses, and are detected in different cell types [35]. In resting human cells the predominant nuclear localization of HSF1 before and after the heat shock has been reported [36], and our analysis suggests that HSF1 partially remains in the cytoplasm of the infected macrophages (Fig. 5). Active translocation of several proteins from the nucleus to serve as transcription factors was already found for some viruses, which conduce their life cycle in the cytoplasm. Recent findings provide evidence that YY1 translocates into the cytoplasm of the vaccinia virus infected cells to serve as an activator of one of vaccinia late genes [37]. The factor is recruited to the cytoplasm of the vaccinia virus-infected macrophages through an exportin-1 system, sensitive to leptomycin B [38].

The vaccinia virus infection resulted in massive recruitment of the HSE-binding activity in the investigated cells (Fig. 3). Surprisingly, only a small fraction of this activity was recognised by the anti-HSF-1-specific antibody, and the 'supershifted' fraction was constitutive (Fig. 3A). We speculate that the observed HSE-binding activity contains HSF1, but the most of its epitopes were obscured by the virus-induced chaperones, which accumulated in the nuclei of the infected macrophages in abundant amounts. The speculation is supported by the results presented in Fig. 6B, which demonstrate the preferential nuclear accumulation of HSP70 and the lack of cytoplasmic accumulation of HSP70 (Fig. 6B) after the vaccinia virus infection. Consequently, the observed HSE-binding activity was much stronger in the nuclear extracts (lane 1) than in the whole cell extracts (lanes 2–4) (Fig. 3A). The similar HSE-binding activity was observed in the extracts from the vaccinia-infected or the heat shocked HepG2 cells (Fig. 3B). It is possible that mostly HSP70 and HSP90 recognise and strongly bind the preformed HSF1-HSE complexes *in vitro *[39]. It seems that the massive nuclear accumulation of stress chaperones is characteristic for the vaccinia virus-infected cells.

MDMs used in our study, survived the VV infection although the virus-induced stress reaction developed accordingly to the infecting dose (Fig. 1). The cytoprotective role of the stress seems evident, since the infected macrophages effectively accumulated different mRNA species for at least 4 days post infection. Routine fluorescent microscopic examination revealed no propidium iodide permeability of the infected cells (not shown). We have previously described that human peripheral blood monocytes retain viability following the infection with low doses of VV [4]. Moreover, in the same experimental conditions HSP70 protected the monocytes against *Staphylococcus aureus*-induced apoptosis [40]. Apparently, the challenge by *S. aureus *might be less tolerable for monocytes/macrophages than the one caused by vaccinia virus. It is due to the staphylococcal α-toxin, which is known to initiate this type of monocyte apoptosis [41]. The poxvirus-induced cytoprotection seems to be much more effective than the other types of stress reaction. Such conclusion can be drawn from the predominant nuclear localization of HSP70 induced in human cells by the vaccinia virus. The predominant nuclear localization of HSP70 was also observed in the respiratory syncytial virus infected cells [42]. The nuclear stress reaction has been found essential for protection also against hypoxia and oxidative stress [43]. Viral antiapoptotic proteins like the recently discovered F1L [44] certainly act in concert with Bcl-2 [45] and stress-induced chaperones to prolong lifespan of the infected cells. Little is known about the impact of pathogen-induced monocyte/macrophage apoptosis in immune system. Persistence of professional immune cells harboring intracellular pathogen in a circulation or a lymph tissue seems detrimental for immunity for at least two reasons: firstly, the immune response is deregulated by cytokines and impaired antigen presentation; secondly, the cells were proposed to serve as virus incubators [46].

Cells of monocytic lineage have recently been recognised as a crucial model to study virus-host interactions due to unique capability of these cells to cross-present endocytosed antigens, especially in the context of chaperone proteins [47]. Further understanding of the heat shock response during the vaccinia virus infection may improve strategies of application of vaccinia genome in recombinant gene expression, vaccination and gene therapy.

## Declaration of competing interests

The author(s) declare that they have no competing interests.

## Authors' contributions

AK carried out monocyte isolation and culture and participated in the immunoassays, KG participated in the design of the study and carried out RT-PCR analysis, KS participated in the gel shift analysis and carried out immunocytochemical analyses, JD participated in the immunoassays, HR conceived the study, participated in its design and coordination, participated in the gel shift analysis and drafted the manuscript. All authors read and approved the final manuscript.
